# Live large flatworm in intestine

**DOI:** 10.11604/pamj.2021.39.209.30820

**Published:** 2021-07-22

**Authors:** Medhavi Vivek Joshi, Chaitanya Ajay Kulkarni

**Affiliations:** 1 Ravi Nair Physiotherapy College, Datta Meghe Institute of Medical Sciences, Sawangi, Wardha, Maharashtra, India

**Keywords:** Flatworm, endoscopy, cholangiopancreatography

## Image in medicine

A 40-year male presented to the emergency department with the complaints of fatigue that had progressively worsened over the period of 3 months. He had recently emigrated from Mexico, where he worked on a farm and eaten raw watercress. Clinical examination revels pallor of oral mucosa. Laboratory findings showed an increased in absolute eosinophil count of 1400 cells per cubic millimeter (reference range 0-400). Ultrasonography of the abdomen reveled intrahepatic biliary ductal dilatation and magnetic resonance cholangiopancreatography revealed a hilar stricture. Endoscopic retrograde cholangiopancreatography was then performed and large flatworm were visualized emerging from major duodenal papilla.

**Figure 1:**
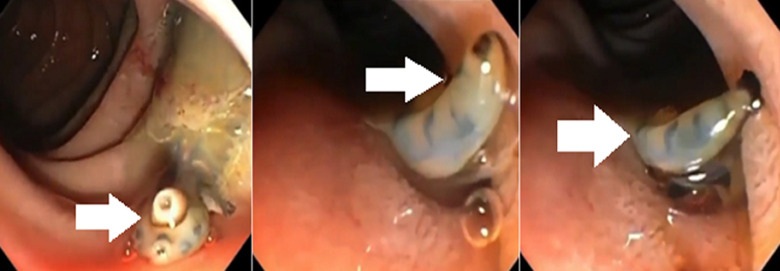
lively flatworm in the duodenal papilla moving on the surface of the intestine

